# HLA class I supertypes: a revised and updated classification

**DOI:** 10.1186/1471-2172-9-1

**Published:** 2008-01-22

**Authors:** John Sidney, Bjoern Peters, Nicole Frahm, Christian Brander, Alessandro Sette

**Affiliations:** 1 Division of Vaccine Discovery, The La Jolla Institute for Allergy and Immunology, 9420 Athena Circle, La Jolla, CA 92037, USA; 2 Partners AIDS Research Center, Massachusetts General Hospital, Harvard Medical School, 149 13 Street, Charlestown, MA 02129, USA

## Abstract

**Background:**

Class I major histocompatibility complex (MHC) molecules bind, and present to T cells, short peptides derived from intracellular processing of proteins. The peptide repertoire of a specific molecule is to a large extent determined by the molecular structure accommodating so-called main anchor positions of the presented peptide. These receptors are extremely polymorphic, and much of the polymorphism influences the peptide-binding repertoire. However, despite this polymorphism, class I molecules can be clustered into sets of molecules that bind largely overlapping peptide repertoires. Almost a decade ago we introduced this concept of clustering human leukocyte antigen (HLA) alleles and defined nine different groups, denominated as supertypes, on the basis of their main anchor specificity. The utility of this original supertype classification, as well several other subsequent arrangements derived by others, has been demonstrated in a large number of epitope identification studies.

**Results:**

Following our original approach, in the present report we provide an updated classification of HLA-A and -B class I alleles into supertypes. The present analysis incorporates the large amount of class I MHC binding data and sequence information that has become available in the last decade. As a result, over 80% of the 945 different HLA-A and -B alleles examined to date can be assigned to one of the original nine supertypes. A few alleles are expected to be associated with repertoires that overlap multiple supertypes. Interestingly, the current analysis did not identify any additional supertype specificities.

**Conclusion:**

As a result of this updated analysis, HLA supertype associations have been defined for over 750 different HLA-A and -B alleles. This information is expected to facilitate epitope identification and vaccine design studies, as well as investigations into disease association and correlates of immunity. In addition, the approach utilized has been made more transparent, allowing others to utilize the classification approach going forward.

## Background

Class I major histocompatibility complex (MHC) molecules bind short peptides derived from the processing of proteins, and present them on the cell surface for T cell scrutiny. Functional MHC molecules are made of a heavy (α) chain and a β2-microglobulin chain. Peptide binding by class I molecules is accomplished by interaction of the peptide amino acid side chains with discrete pockets within the peptide-binding groove of the MHC molecule formed by the α1 and α2 domains of the heavy chain. Typically, in the case of human leukocyte antigen (HLA) class I, the main binding energy is provided by the interaction of residues in position 2 and the C-terminus of the peptide with the B and F binding pockets of the MHC molecule, respectively [1-8], although side chains throughout the ligand can have a positive or negative influence on binding capacity. The common chemical specificity of the peptide side chains amongst ligands bound by a specific MHC molecule is termed the binding motif [9].

MHC molecules are extremely polymorphic, and over a thousand allelic variants have already been described at the class I A and B loci. Most of the polymorphism is located in the peptide-binding region, and as a result each variant is believed to bind a unique repertoire of peptide ligands. Despite this polymorphism, HLA class I molecules can be clustered into groups, designated as supertypes, representing sets of molecules that share largely overlapping peptide binding specificity. Each supertype can be described by a supermotif that reflects the broad main anchor motif recognized by molecules within the corresponding supertype. For example, molecules of the A2-supertype share specificity for peptides with aliphatic hydrophobic residues in position 2 and at the C-terminus, while A3-supertype molecules recognize peptides with small or aliphatic residues in position 2 and basic residues at the C-terminus.

The first supertypes were described in the mid-1990's by our group [10]. Using motifs derived from binding data or the sequencing of endogenously bound ligands, along with simple structural analyses, nine different supertypes were defined [11,12]. Initial peptide binding studies allowed the identification of several peptides with degenerate binding capacity for the A2- [13-15], A3- [16-18] and B7-supertypes [19,20]. In subsequent years, additional binding data have been published to confirm the B44- [21], A1- and A24-supertypes [22].

Over time, a number of different and more sophisticated computational approaches have been implemented by others to classify HLA class I alleles into clusters or supertypes [23-32]. While in general agreement, additional supertypes, representing either sub-clusters of the original nine or completely novel groupings, have been proposed [23,32]. Despite differences in various classification schemes, the concept of HLA supertypes has been effectively used to characterize and identify promiscuously recognized T cell epitopes from a variety of different disease targets, including measles-mumps-rubella [33], SARS [34], EBV [35,36], HIV [37-41], Kaposi' Sarcoma Associated Human Herpesvirus [42,43], HCV [16,44], HBV [45,46], HPV [47], influenza [48], P. falciparum [49-52], LCMV [53], Lassa virus [54], F. tularensis [55], vaccinia [56-58], and also cancer antigens [59-68]. Supertypes have also been utilized as a component in several approaches and algorithms designed for predicting peptide candidates with degenerate HLA class I binding capacity [69-77]. Finally, supertypes have also been examined as a variable in studies of disease association, rates of susceptibility, and outcome [37,78-86].

The original classification proposed by Sette and Sidney [12] comprised about 100 different class I MHC alleles. However, over the last 10 years, substantially more binding data has been generated, and through the efforts of several seminal MHC-related databases, including SYFPEITHI [87], HLA Ligand [88], FIMM [89] and MHCBN [90], MHC binding motif information is readily accessible. Up-to-date compilations of MHC sequence data are also readily available in the IMGT database [91]. Herein, we analyze the complete list of alleles available through IMGT (release 2.9) using a simple approach largely based on compilation of published motifs, binding data, and analyses of shared repertoires of binding peptides, in conjunction with clustering based on the primary sequence of the B and F peptide binding pockets. As a result, we now provide updated supertype assignments, with new assignments for about 700 different HLA-A and -B alleles. This will permit the use of our original classification scheme based on current data and its comparison to alternative supertype classification methodologies developed since then.

## Results

### MHC sequences and peptide binding pockets

α1 and 2 chain residues comprising the B and F peptide-binding pockets of 945 HLA A and B class I molecules were extracted and aligned. Based on crystallographic studies [1,4,6,8], residues 7, 9, 24, 34, 45, 63, 66, 67, 70, and 99 were considered as comprising the B pocket, which engages the peptide residue in position 2. The residues comprising the F pocket, which engages the peptide C-terminal residue, were defined as 74, 77, 80, 81, 84, 95, 97, 114, 116, 123, 133, 143, 146, and 147. Further, for the B pocket we defined the subset of residues 9, 45, 63, 66, 67, 70, and 99 as key residues. In the F pocket, the key residues are those in positions 77, 80, 81 and 116.

### Pocket chemical specificity

Next, we defined the broad chemical specificity associated with the different B and F pockets. Table 1 summarizes the amino acid residues generally associated with each particular description of binding specificity. The particular set of residues associated with each use of a descriptor may vary with context. While these descriptions largely follow classical textbook definitions, they also consider our own historical use and experience with peptide binding studies. For example, in our analyses we often consider the polar residue Q along with more classically defined aliphatic residues based on observed behavior in MHC peptide binding studies [15,92,93]. It is also important to note that some residues are assigned to multiple categories, as their chemical specificity is compatible with different emphases. For example, L would be considered as both aliphatic and hydrophobic. The chemical specificities defining each supertype are listed in Table 2.

**Table 1:** Physiochemical functionality of peptide side chains.

**Pocket specificity**	**Associated amino acid residues**
Acidic	DE
Aliphatic	LIVMQ
Aromatic	FWY
Aromatic and aliphatic	FWYLIVMQ
Aromatic and basic	YRK
Aromatic and large hydrophobic	FWYLIM
Basic	RHK
Hydrophobic	LIVMFWYA
Large hydrophobic	FLIM
Proline	P
Small	AST
Small and aliphatic	ATSVLIMQ
Small hydrophobic	AV
Small, aliphatic and aromatic	ASTVLIMQFWY

**Table 2:** HLA supertype specificity descriptions.

**Supertype(s)**	**B pocket specificity**	**F pocket specificity**
A01	Small and aliphatic	Aromatic and large hydrophobic
A01 A03	Small and aliphatic	Aromatic and basic
A01 A24	Small, aliphatic and aromatic	Aromatic and large hydrophobic
A02	Small and aliphatic	Aliphatic and small hydrophobic
A03	Small and aliphatic	Basic
A24	Aromatic and aliphatic	Aromatic, aliphatic and hydrophobic
B07	Proline	Aromatic, aliphatic and hydrophobic
B08	Undefined	Aromatic, aliphatic and hydrophobic
B27	Basic	Aromatic, aliphatic, basic and hydrophobic
B44	Acidic	Aromatic, aliphatic and hydrophobic
B58	Small	Aromatic, aliphatic and hydrophobic
B62	Aliphatic	Aromatic, aliphatic and hydrophobic

### Peptide-binding motif-based supertype assignments

As a starting point, we have largely utilized the nine supertype designations derived previously [11,12], with a few exceptions. These exceptions entail specificities that appear to be compatible with multiple supertypes. More specifically, we recognize that some alleles have repertoires overlapping both the A01 and A03 supertypes (e.g., A*3001; see Hamdahl et al., IEDB submission 1000945 [94-96]), and others with the A01- and A24-supertypes (e.g., A*2902 [22]). Also, because it utilizes a non-canonical main anchor spacing, and as a result appears to have a repertoire that overlaps with other specificities, we have presently separated HLA B*0801 and other alleles sharing sequence and serological antigen similarities, as a separate cluster. While these B*08 alleles utilize a unique mode of peptide binding, it is likely that in most cases their repertoires overlap significantly with other supertypes, especially the B07-supertype (Sidney, Frahm, Brander and Sette, unpublished observations). As a result, we have not defined this a separate supertype.

Next, the available peptide-binding motifs were compiled. In total, 88 different class I motifs were identified. Motif information was derived from our own peptide-binding studies, or from the published scientific literature as compiled in the SYFPEITHI database [87]. The basis for each motif assignment is listed in Tables 3 and 4 for HLA-A and -B alleles, respectively. Corresponding supertype associations were assigned based on the criteria listed in Table 2.

**Table 3:** HLA-A motif/structure reference panel alleles.

**Allele**	**Basis for motif**	**Supertype association**
A*0101	Binding assay	A01
A*0201	Binding assay	A02
A*0202	Binding assay	A02
A*0203	Binding assay	A02
A*0204	Pool sequencing/ligand elution	A02
A*0205	Binding assay	A02
A*0206	Binding assay	A02
A*0207	Binding assay	A02
A*0214	Pool sequencing/ligand elution	A02
A*0217	Pool sequencing/ligand elution	A02
A*0301	Binding assay	A03
A*1101	Binding assay	A03
A*2301	Binding assay	A24
A*2402	Binding assay	A24
A*2601	Binding assay	A01
A*2602	Pool sequencing/ligand elution	A01
A*2603	Pool sequencing/ligand elution	A01
A*2902	Binding assay	A01 A24
A*3001	Binding assay	A01 A03
A*3002	Binding assay	A01
A*3003	Pool sequencing/ligand elution	A01
A*3004	Pool sequencing/ligand elution	A01
A*3101	Binding assay	A03
A*3201	Binding assay	A01
A*3301	Binding assay	A03
A*3303	Published motif	A03
A*6601	Pool sequencing/ligand elution	A03
A*6801	Binding assay	A03
A*6802	Binding assay	A02
A*6901	Pool sequencing/ligand elution	A02
A*7401	Binding assay	A03

**Table 4:** HLA-B motif/structure reference panel alleles.

**Allele**	**Basis for motif**	**Supertype association**
B*0702	Binding assay	B07
B*0703	Pool sequencing/ligand elution	B07
B*0705	Pool sequencing/ligand elution	B07
B*0801	Binding assay	B08
B*0802	Pool sequencing/ligand elution	B08
B*1402	Pool sequencing/ligand elution	B27
B*1501	Binding assay	B62
B*1502	Pool sequencing/ligand elution	B62
B*1503	Binding assay	B27
B*1508	Pool sequencing/ligand elution	B07
B*1509	Pool sequencing/ligand elution	B27
B*1510	Pool sequencing/ligand elution	B27
B*1512	Pool sequencing/ligand elution	B62
B*1513	Pool sequencing/ligand elution	B62
B*1516	Pool sequencing/ligand elution	B58
B*1517	Pool sequencing/ligand elution	B58
B*1518	Pool sequencing/ligand elution	B27
B*1801	Binding assay	B44
B*2702	Pool sequencing/ligand elution	B27
B*2703	Pool sequencing/ligand elution	B27
B*2704	Pool sequencing/ligand elution	B27
B*2705	Binding assay	B27
B*2706	Pool sequencing/ligand elution	B27
B*2707	Pool sequencing/ligand elution	B27
B*2709	Pool sequencing/ligand elution	B27
B*3501	Binding assay	B07
B*3503	Pool sequencing/ligand elution	B07
B*3701	Pool sequencing/ligand elution	B44
B*3801	Pool sequencing/ligand elution	B27
B*3901	Pool sequencing/ligand elution	B27
B*3902	Pool sequencing/ligand elution	B27
B*3909	Pool sequencing/ligand elution	B27
B*4001	Binding assay	B44
B*4002	Binding assay	B44
B*4006	Pool sequencing/ligand elution	B44
B*4201	Binding assay	B07
B*4402	Binding assay	B44
B*4403	Binding assay	B44
B*4501	Binding assay	B44
B*4601	Pool sequencing/ligand elution	B62
B*4801	Pool sequencing/ligand elution	B27
B*5101	Binding assay	B07
B*5102	Pool sequencing/ligand elution	B07
B*5103	Pool sequencing/ligand elution	B07
B*5201	Pool sequencing/ligand elution	B62
B*5301	Binding assay	B07
B*5401	Binding assay	B07
B*5501	Pool sequencing/ligand elution	B07
B*5502	Pool sequencing/ligand elution	B07
B*5601	Pool sequencing/ligand elution	B07
B*5701	Binding assay	B58
B*5702	Pool sequencing/ligand elution	B58
B*5801	Binding assay	B58
B*5802	Binding assay	B58
B*6701	Pool sequencing/ligand elution	B07
B*7301	Pool sequencing/ligand elution	B27
B*7801	Pool sequencing/ligand elution	B07

### Pocket analysis and supertype assignments

The amino acids comprising the B and F peptide binding pockets were compiled for all alleles for which complete sequence information was available. The positions of specific MHC residues forming the corresponding pockets are listed above. A reference panel of B and F pockets was generated to include all alleles for which the MHC-peptide binding specificity has been defined (see Tables 3 and 4). These B and F pocket structures, along with the associated alleles and corresponding binding specificity are shown in Tables 5 and 6, respectively.

**Table 5:** B pocket structures of reference panel alleles.

**Residues**^1^	**Key residues**	**Reference panel allele(s)**	**Associated supertype(s)**	**Specificity**
YDSVENIFNY	DENIFNY	B*0801-2	B08	Undefined
YFAVMEKVHC	FMEKVHC	A*0207	A02	Small and aliphatic
YFAVMEKVHF	FMEKVHF	A*0217	A02	Small and aliphatic
YFAVMEKVHY	FMEKVHY	A*0201-4	A02	Small and aliphatic
YFAVMENMHY	FMENMHY	A*0101	A01	Small and aliphatic
YFAVMENVHY	FMENVHY	A*3201, A*7401	A01 A03	Small and aliphatic
YFAVMENVQY	FMENVQY	A*0301	A03	Small and aliphatic
YHSVTEISNS	HTEISNS	B*3701	B44	Acidic
YHSVTNISNY	HTNISNY	B*1801	B44	Acidic
YHTVEEICKY	HEEICKY	B*2702-7, B*2709	B27	Basic
YHTVENICKY	HENICKY	B*7301	B27	Basic
YHTVKEISNY	HKEISNY	B*4001-2, B*4006, B*4501	B44	Acidic
YSAVMEKVHF	SMEKVHF	A*2301, A*2402	A24	Aromatic and aliphatic
YSAVMENVHY	SMENVHY	A*3002-4	A01	Small and aliphatic
YSAVMENVQY	SMENVQY	A*3001	A01 A03	Small and aliphatic
YTAVMENVHY	TMENVHY	A*3101	A03	Small and aliphatic
YTAVMNNVHY	TMNNVHY	A*3301, A*3303	A03	Small and aliphatic
YTAVMQNVQY	TMQNVQY	A*2902	A01 A24	Small, aliphatic and aromatic
YYAVENIYQY	YENIYQY	B*5501-2, B*5601	B07	Proline
YYAVGNIYQY	YGNIYQY	B*5401	B07	Proline
YYAVMEISNY	YMEISNY	B*1501, B*1512	B62	Aliphatic
YYAVMEKVHY	YMEKVHY	A*0205, A*0206, A*0214	A02	Small and aliphatic
YYAVMEKYQY	YMEKYQY	B*4601	B62	Aliphatic
YYAVMENMSY	YMENMSY	B*1516-17, B*5701-2	B58	Small
YYAVMENVQY	YMENVQY	A*1101	A03	Small and aliphatic
YYAVMNIFNY	YMNIFNY	B*1508	B07	Proline
YYAVMNISNY	YMNISNY	B*1502, B*1513	B62	Aliphatic
YYAVMNNVHY	YMNNVHY	A*2601-3	A01	Small and aliphatic
YYAVMNNVQY	YMNNVQY	A*6601, A*6801-2, A*6901	A02 A03	Small and aliphatic
YYAVTEISNY	YTEISNY	B*5201	B62	Aliphatic
YYAVTENMSY	YTENMSY	B*5801-2	B58	Small
YYAVTNIFNY	YTNIFNY	B*3501, B*3503, B*5101-3, B*5301, B*7801	B07	Proline
YYSVEEISNY	YEEISNY	B*1503, B*3902, B*4801	B27	Basic
YYSVENICNS	YENICNS	B*3909	B27	Basic
YYSVENICNY	YENICNY	B*1402, B*1509-10, B*1518, B*3801, B*3901	B27	Basic
YYSVENIYNY	YENIYNY	B*0703	B07	Proline
YYSVENIYQY	YENIYQY	B*0702, B*0705, B*4201, B*6701	B07	Proline
YYTVKEISNY	YKEISNY	B*4402-3	B44	Acidic

1. Based on data presented in Saper et al, and Madden (see Methods), the MHC residues utilized as constituting the B pocket are: 7, 9, 24, 34, 45, 63, 66, 67, 70, and 99. Key residues are those in positions 9, 45, 63, 66, 67, 70, and 99.

**Table 6:** F pocket structures of reference panel alleles.

**Residues**^1^	**Key residues**^2^	**Reference panel allele(s)**	**Associated supertype(s)**	**Specificity**
DDTLYIIEHYWTKW	DTLH	A*3001	A01 A03	Aromatic and basic
DDTLYIIRDYWTKW	DTLD	A*0301, A*1101	A03	Basic
DDTLYIMQDYWTKW	DTLD	A*3101, A*3301, A*3303, A*7401	A03	Basic
DDTLYIMRDYWTKW	DTLD	A*6801	A03	Basic
DDTLYIRHYYWTKW	DTLY	A*6802	A02	Aliphatic
DDTLYIRQDYWTKW	DTLD	A*6601	A03	Basic
DDTLYLNHDYWTKW	DTLD	B*2703, B*2705	B27	Hydrophobic and basic
DDTLYLNHHYWTKW	DTLH	B*2709	B27	Large hydrophobic
DDTLYLSNYYWTKW	DTLY	B*2707	B27	Large hydrophobic
DDTLYVRHYYWTKW	DTLY	A*6901	A02	Aliphatic
DGNLYWTNFYWTKW	GNLF	B*7301	B27	Large hydrophobic
DNIAYLMHYYWTKW	NIAY	A*2301, A*2402	A24	Large hydrophobic
DNIAYLNHDYWTKW	NIAD	B*2702	B27	Large hydrophobic
DNTAYLSNYYWTKW	NTAY	B*0802	B08	Hydrophobic
DNTLYIIEHYWTKW	NTLH	A*3002-4	A01	Aromatic
DNTLYIIRDYWTKW	NTLD	A*0101	A01	Aromatic
DNTLYIMRDYWTKW	NTLD	A*2902	A01 A24	Aromatic and large hydrophobic
DNTLYIRQDYWTKW	NTLD	A*2601	A01	Aromatic
DNTLYIRQNYWTKW	NTLN	A*2602	A01	Aromatic
DSIAYIMQDYWTKW	SIAD	A*3201	A01	Aromatic
DSNLYLRDSYWTKW	SNLS	B*4601	B62	Large hydrophobic
DSNLYLRNFYWTKW	SNLF	B*3901-2, B*3909, B*6701	B07 B27	Hydrophobic
DSNLYLSDYYWTKW	SNLY	B*0702-3	B07	Hydrophobic
DSNLYLSNYYWTKW	SNLY	B*0705, B*0801, B*4201	B07	Hydrophobic
DSNLYLWNFYWTKW	SNLF	B*1402	B27	Hydrophobic
DSNLYWTNLYWTKW	SNLL	B*5401, B*5501-2, B*5601	B07	Small hydrophobic
DSNLYWTNYYWTKW	SNLY	B*7801	B07	Hydrophobic
DSTLYLNDYYWTKW	STLY	B*2706	B27	Large hydrophobic
DSTLYLNHDYWTKW	STLD	B*2704	B27	Large hydrophobic
HDTLYIRQDYWTKW	DTLD	A*2603	A01	Aromatic
HDTLYLMHYYWTKW	DTLY	A*0217	A02	Aliphatic
HDTLYLRHYYWTKW	DTLY	A*0202, A*0205, A*0214	A02	Aliphatic
HDTLYVMHYYWTKW	DTLY	A*0204	A02	Aliphatic
HDTLYVRHYYWTKW	DTLY	A*0201, A*0203, A*0206-7	A02	Aliphatic
YDTLYIRNFYWTKW	DTLF	B*3701	B44	Hydrophobic
YNIAYIRDSYWTKW	NIAS	B*1513, B*5301, B*5801	B07 B58 B62	Large hydrophobic
YNIAYIVDSYWTKW	NIAS	B*5701	B58	Large hydrophobic
YNIAYIVNYYWTKW	NIAY	B*5702	B58	Large hydrophobic
YNIAYLRHDYWTKW	NIAD	B*1517	B58	Large hydrophobic
YNIAYLRNFYWTKW	NIAF	B*3801	B27	Large hydrophobic
YNIAYLWDSYWTKW	NIAS	B*5802	B58	Large hydrophobic
YNIAYWRDSYWTKW	NIAS	B*1516	B58	Large hydrophobic
YNIAYWTNYYWTKW	NIAY	B*5101-3, B*5201	B07 B62	Large hydrophobic
YNTAYIRDDYWTKW	NTAD	B*4402-3	B44	Hydrophobic
YSNLYIRDFYWTKW	SNLF	B*3503	B07	Hydrophobic
YSNLYIRDSYWTKW	SNLS	B*1502, B*3501	B07 B62	Hydrophobic
YSNLYLRDSYWTKW	SNLS	B*1501, B*1503, B*1508, B*1512, B*1518, B*1801	B07 B27 B44 B62	Large hydrophobic
YSNLYLRDYYWTKW	SNLY	B*1510	B27	Hydrophobic
YSNLYLRNYYWSKL	SNLY	B*4001	B44	Hydrophobic
YSNLYLRNYYWTKW	SNLY	B*1509	B27	Hydrophobic
YSNLYLSNYYWSKL	SNLY	B*4801	B27	Hydrophobic
YSNLYLSNYYWTKW	SNLY	B*4002	B44	Hydrophobic
YSNLYWRNLYWTKW	SNLL	B*4501	B44	Small hydrophobic
YSNLYWTNYYWTKW	SNLY	B*4006	B44	Hydrophobic

1. Based on data presented in Saper et al, and Madden (see Methods), the MHC residues utilized as constituting the F pocket are: 74, 77, 80, 81, 84, 95, 97, 114, 116, 123, 133, 143, 146, and 147. Key residues are those in positions 77, 80, 81, and 116.

2. The key residue pockets DTLD, DTLH and DTLY are each associated with two different, although overlapping, specificity descriptions. In each case, one description is associated with HLA B27 alleles, and in the other with HLA A alleles. In assigning key residue matches, matches were assigned on the basis of corresponding locus.

Next, the B and F pocket structures of alleles whose peptide binding specificity was unknown were compared against the sequences in the reference panel. For each case, an attempt was made to find an exact match with the full set of residues in the corresponding pocket. If a match was identified, the allele was assigned the associated specificity shown in Tables 5 and 6. If an exact match could not be found, then a match with a key residue sequence was attempted. If no match was identified, the allele was considered unassigned. For each allele where a match for the full or, secondarily, key residue sequence could be identified for both the B and F pockets with any of the alleles indicated in Tables 3 and 4, a corresponding HLA supertype was assigned.

**Figure 1:**
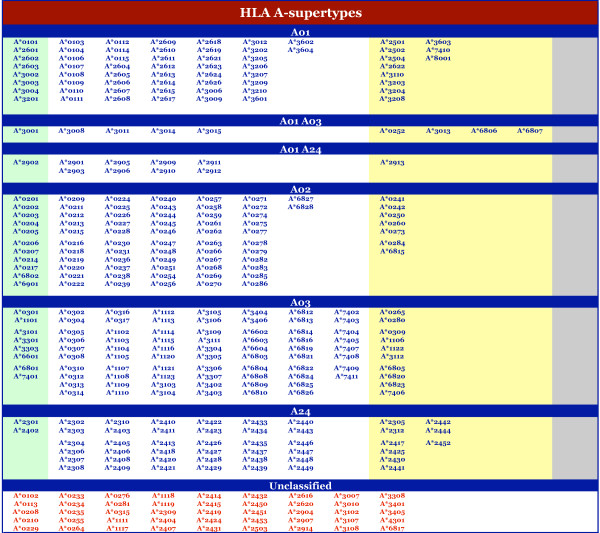
**Supertype classification of HLA-A alleles**. The alleles associated with each HLA-A supertype, multiple supertypes, or that are unclassified, are shown. Under each supertype, alleles are group (by color) on the basis of the stringency of selection: experimentally established motif (i.e., reference panel) (green), exact match(es) in the B and F pockets (white), one exact and one key residue pocket match (yellow), key residue match(es) at B and F pockets (grey). Alleles with no match at one or both pockets are listed with red font.

**Figure 2:**
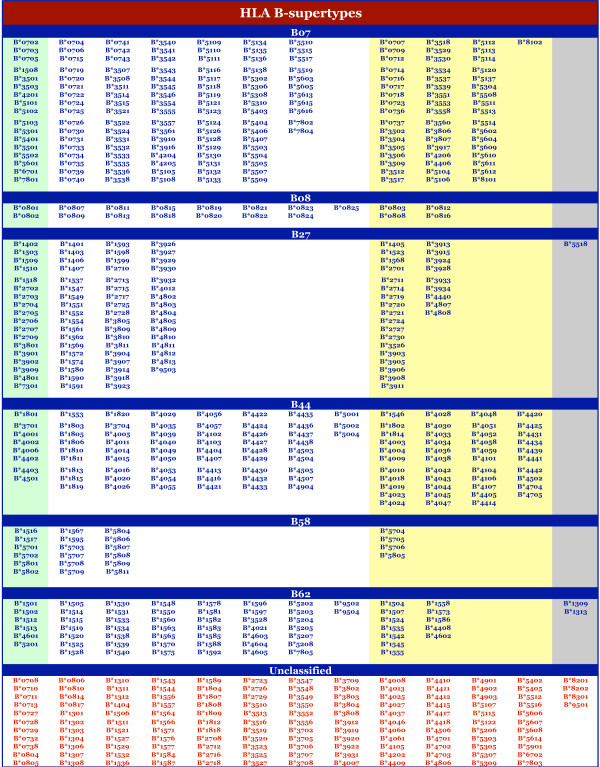
**Supertype classification of HLA-B alleles**. The alleles associated with each HLA-B supertype, multiple supertypes, or that are unclassified, are shown. Under each supertype, alleles are grouped (by color) on the basis of the stringency of selection as described in the legend to Figure 1.

Of the 945 sequences analyzed, matches at both B and F pockets were found for 764 (80.8%) (Table 7). Notably, for the majority (57%) of alleles not in the reference panel full sequence matches were identified at both the B and F pockets. Figures 1 and 2 indicate the alleles associated with each HLA-A or -B supertype, respectively. Conversely, as an index, Additional file 1 provides the supertype assignment for each of the 945 HLA-A and -B alleles examined, along with their respective B and F pocket structures.

**Table 7:** Quantification of supertype assignments.

		**Classification criteria**				
		
**Supertype**	**(n)**	**Binding assay**	**Pool sequencing or ligand elution**	**Full sequence match at B and F**	**1 full and 1 key sequence match**	**Key residue match at B and F**
A01	61	4	4	42	11	0
A01 A03	9	1	0	4	4	0
A01 A24	10	1	0	8	1	0
A02	75	7	4	57	7	0
A03	86	6	2	68	10	0
A24	47	2	0	36	9	0
B07	165	6	11	96	52	0
B08	19	1	1	13	4	0
B27	98	2	16	52	27	1
B44	108	6	2	57	43	0
B58	22	3	3	12	4	0
B62	64	1	5	44	12	2
Unassigned	181	-	-	-	-	-

Total	945	40	48	489	184	3

At the HLA-A locus, alleles were fairly evenly distributed amongst the four supertypes we have defined. Considering the alleles with broad, or dual, specificity (i.e., those assigned as A01–A03 or A01–A24), the minimum was 57 alleles for A24, and the maximum was 95 alleles for A03. 75 alleles were assigned to the A02 supertype, and 80 to the A01 supertype. At the HLA-B locus, the B07-supertype is the largest, with 165 members. 19 alleles were associated with the B08 pattern. As noted above, we hypothesize that alleles in this cluster will likely cross-react with other supertypes (especially B07), so we do not consider this group as a distinct supertype. The B58 supertype was assigned only 22 alleles, making it the smallest supertype.

### Novel supertypes could not be identified, but several alleles have specificities spanning two different supertypes

From Tables 5 and 6, it is evident that not all possible combinations of B and F pocket specificities are present within the reference panel. For example, there are no alleles associated with a preference for acidic residues in position 2 and basic residues at the C-terminus. Hence, we were interested to see if combinations not found in the original supertype assignments were revealed in the present analysis. Interestingly, we did not identify any new combinations. It is possible that new specificities will be identified from amongst the set of 181 alleles for which pocket matches could not be obtained. However, we also note that in the majority of these cases, matches could be made if one allowed a single conservative residue change (e.g., E to D). Thus, based on the analyses done to date, it would appear that the set of supertype specificities currently identified will cover virtually all HLA-A and -B class I MHC alleles.

In addition to A*2902 and A*3001, the present analysis has identified 17 alleles expected to have a specificity overlapping two supertypes. Nine alleles matched pocket preference patterns that would be compatible with both the A01- and A03-supertypes, and another 10 with the A01- and A24-supertypes. In the former case, the association was established on the basis of pocket matches with A*3001. Not surprisingly, several, but not all, of the alleles assigned the A01–A03 specificity also share the A30 antigen. The A01–A24 cross-reactivity pattern was established on the basis of pocket matches with A*2902. All of the alleles associated with the A01–A24 pattern also shared the A29 antigen.

### Unexpected supertype assignments

Typically, the supertype assignment for a particular allele follows the predominant assignment for other alleles sharing the same serological antigen. With this in mind, we did identify several instances where the current classification differed from what was expected based on serology or previous classification (Table 8). Of these, the more striking ones involve alleles for which the assigned supertype represents a non-conservative change in the binding specificity from what would have been expected. For example, A*0265 and A*0280, which are associated with the A02 serological antigen, and thus might be expected to have an F pocket specificity for hydrophobic/aliphatic residues, were found to have an F pocket matching that of A03 supertype alleles, which have a specificity for positively charged residues. Similarly, B*4012 and B*4440 were found to have B27 supertype B pockets, associated with a preference for positively charged residues, when a B44 supertype specificity for acidic residues might have been expected.

**Table 8:** Unexpected and/or revised supertype assignments.

	**Supertype assignment**	
	
**Allele**	**Expected**^1^**or previous**	**Current**
A*0252	A02	A01 A03
A*0265	A02	A03
A*0280	A02	A03
A*3001	A24	A01 A03
A*3002	A24	A01
A*3003	A24	A01
A*3110	A03	A01
A*7410	A03	A01
B*3528	B07	B62
B*3806	B27	B07
B*3807	B27	B07
B*3910	B27	B07
B*3916	B27	B07
B*3917	B27	B07
B*4012	B44	B27
B*4021	B44	B62
B*4406	B44	B07
B*4408	B44	B62
B*4440	B44	B27
B*5518	B07	B27

1. Expected based on serological association.

As noted above, in the present analysis, and contrary to what was done in the original analysis, we did not make supertype assignments for alleles without an exact or key match by considering conservative substitutions. As a result of this more conservative approach, we now no longer assign supertypes to some alleles previously given a supertype designation (Table 9). In the majority of these cases, however, the original designation would still seem to be a reasonable assumption.

**Table 9:** Alleles reclassified as "unassigned".

**Allele**	**Previous assignment**	**B pocket match**	**F pocket match**	**Comments**
A*0102	A01	-	A01 full	Single difference with A*0101 (F to S) at position 9 in B pocket is non-conservative.
A*2404	A24	A24 full	-	F pocket key sequence is similar to A02 and B27 (hydrophobic), so A24 is still likely.
A*3401	A03	-	A03 full	N/K in 63/66 are relatively unique, otherwise similar to A03 in the B pocket.
A*4301	A01	-	A01 full	Q to N in 63 is only difference with A*2601, so A01 is still likely.
B*1301	B62	B62 key	-	Unusual F pocket.
B*1302	B62	B62 key	-	Unusual F pocket.
B*1506	B62	-	B07 B27 B44 B62 full	Single conservative change (Y to F) at position 99 of B pocket, comapred to B*1501; B62 still likely.
B*1521	B62	-	B07 B62 full	S to C change in position 67 is the onlyB pocket difference with B*1501, so B62 is still likely.
B*2708	B27	B27 full	-	Position 77 T to N change is only difference with b*2704, so B27 is still likely.
B*3802	B27	B27 full	-	I to T in position 80 of the F pocket is the only difference with B*3801, so B27 is still likely.
B*4701	B44	-	B27 key	Y to F at position 99 is the only difference with B*4402-3 in the B pocket, so B44 is still likely.
B*4901	B44	B44 full	-	F full pocket is similar to B*3801 (hydrophobic), so B44 is still likely.

## Discussion

In the present study we have attempted to classify almost 1000 HLA-A and -B class I alleles into supertypes. This is nearly a 10-fold increase in the number of alleles compared to our original classification done about a decade ago [12]. Besides providing supertype assignments for considerably more alleles, the present report has attempted to make more transparent how the original "phenomenological" classifications were done. About 80% of the 945 alleles examined were classified into one of the nine supertypes identified previously. Analysis of B and F pocket specificity patterns did not suggest the existence of any novel supertypes.

HLA supertypes do not necessarily demarcate groups of alleles with completely non-overlapping repertoires. A binding repertoire overlapping multiple supertypes has been demonstrated previously, for example, in the cases of A*2902 [22] and A*3001 (see Hamdahl et al., IEDB submission 1000945 [94-96]). In the present study we have identified 17 other alleles that would appear to have specificities that bridge either the A01 and A03 supertypes, or the A01 and A24 supertypes. At the same time, individual peptides can be readily identified that bear a particular supermotif, but that do not bind individual HLA allele members of the supertype, or that bind alleles of other supertypes, even supertypes associated with a different locus. Typically, in the first case, these phenomena result from differences in motif compatibility, perhaps at secondary positions. The second case likely reflects overlap(s) between the supertypes in terms of specificity, although in rare cases binding can be accomplished when no main anchor motif compatibility is apparent.

These observations are exemplified by a large scale analysis of the capacity of a non-redundant set of 252 known EBV and HIV derived epitopes to bind a panel of 30 different HLA class I A and B molecules (Sidney, Frahm, Brander and Sette, unpublished observations). It was found that about 21% of the peptides bearing a specific supermotif bound a given allele in the corresponding supertype with an affinity of 100 nM, or better. By contrast, only in 1% of the cases considered did an allele bind a peptide that did not have the corresponding supermotif. At the same time, it was noted that in the set of peptides utilized 62% (155/252) have motifs associated with 2 or more supertypes. The pattern of binding also followed this general promiscuity. It is also significant to note that when the same library of peptides was examined for recognition in HIV/EBV patients, it was found that ~95% of the epitopes were recognized in individuals not expressing the allele the epitope was originally reported to be restricted by, and the promiscuity more often than not involved an allele outside of the supertype associated with originally described restricting allele [97]. Thus, it is apparent that the lines of demarcation between supertypes can be fuzzy from the perspective of both the allelic specificity and the peptide motif.

Restriction outside or across supertypes can also originate from overlaps in supermotifs (e.g., A02 and B62), or for alleles such as B*0801 which do not utilize the typical P2/Cterminus anchor spacing. B*0801 utilizes P3 and P5, not P2, and as such may be compatible with several supertypes and alleles. Thus, an A02- or A24-supertype epitope cross-reacting with B*0801 is not an example of a motif "failure", but merely reflects the fact that the specific peptide has both motifs.

It is important to emphasize that supertypes are based on MHC binding, and that MHC binding alone is not sufficient criteria for T cell recognition. Indeed, hundreds of examples of peptides that bind with remarkably high affinity, but that are not recognized by T cells, have been reported in the literature. We note that even in the best affinity ranges (i.e., IC50 <10 nM), rarely more than 10% of the peptides can be expected to be recognized [98]. Similarly, binding affinity is not necessarily correlative of frequency of recognition [99]. It is true that the trend is towards the most frequently recognized peptides being also the highest affinity binders [38,50,100], but that is not always the case, and there are clearly cases where the dominant epitope has an IC50 of ~100 nM, while several other non-recognized peptides have affinities in the <10 nM range.

It must also be emphasized that membership of an epitope in a supertype is not sufficient to guarantee its recognition by T cells in the context of different MHC alleles. Peptide binding to MHC is an absolute requirement for an epitope to be recognized by T cells. At the same time, many other factors, including protein expression and processing, as well as T cell repertoire and the specific MHC context, come into play in determining whether a peptide will be an epitope or not, or whether an epitope will be promiscuously recognized within a specific supertype. For example, Goulder and co-workers, studying B7-supertype epitopes, found that differential selection pressure exerted on HIV by CTL targeting identical epitopes, but restricted by distinct HLA alleles from the same supertype, can result in significant functional differences [101]. Macdonald et al., looking at two B44 subtypes described as members of the HLA B44-supertype, reported that a naturally selected dimorphism between the two molecules alters class I structure, peptide repertoire, and T cell recognition [102].

The intent of the current study was to derive an updated classification of HLA class I MHC alleles on the basis of primary anchor specificity. For the vast majority of HLA class I molecules whose binding specificity have been described by crystal structure, pool sequencing or peptide binding studies, the main anchor interactions of the peptide almost invariably involve the MHC B and F pockets, while other pockets likely dictate secondary interactions. This pattern also appears to be true for most macaque and chimpanzee class I alleles studied to date.

There are exceptions, however, and indeed we have not assigned B*08 alleles to a specific supertype in recognition of the fact that these alleles appear to utilize pockets other than the B pocket as a primary anchor contact. For HLA class I molecules in general, the B and F pockets are the most likely main anchor contacts, while other pockets likely dictate secondary interactions. High levels of crossreactivity have been experimentally demonstrated in the case of 6 supertypes for alleles that vary at secondary pockets [15,17,20-22].

By contrast, in the murine system it is well recognized that other pockets are often the important primary peptide contacts [103-109]. Thus, to utilize the classification approach described here in the context of other species, additional pockets may need to be considered. It is also likely that the further parsing or sub-classifying of supertypes on the basis of secondary interactions can be accomplished.

In the case of HLA-B alleles, F pocket specificity is difficult to correlate with a specific sequence, as a diverse pattern of residues appears to be associated with similar binding specificity. Independent of the residues in the F pocket, most HLA-B alleles seem to bind hydrophobic residues. Thus, assignment of B alleles was primarily driven by the specificity exhibited by the B pocket. On the other hand, it is also possible that greater resolution in the F pocket could be achieved as more data become available to discriminate different preference patterns. For example, in the B7 supertype it is apparent that some alleles, like B*3501, prefer large hydrophobic residues, such as Y. Conversely, other B7 supertype alleles, such as B*5401, seem to prefer small hydrophobic residues, such as A, at the C-terminus. While we have noted these subtle differences in preference [20], in practice we have not found that they significantly impact cross-reactivity between the alleles. This perhaps suggests that the C-terminal anchor in some contexts is less important, and that shared secondary preferences can have a stronger influence on degenerate binding capacity than in other cases. At the same time, it may be necessary to consider additional key residues in the analysis of the F pocket. This is exemplified in the cases of A*2603 and A*0301 which have the same key F pocket residues, but which are associated with much different specificity.

The vast majority of HLA-A and -B alleles fall into one of the 9 supertypes we have described. There are likely reasons for this [110], which include evolutionary relationships, but also constraints and limitations inherent in the epitope processing infrastructure. For example, no allele has been identified to date that binds peptides with D, E, Q or P at the C-terminus, which is in congruence with the preferences of both proteasomal cleavage and TAP transport [111], and an observation that has been applied in the rational design of an in vitro test reagent tool (PeptGen) offered as a tool by the Los Alamos HIV Sequence Database [112].

Supertype classification should not be taken to necessarily imply an evolutionary relationship. In some cases this is largely true, as for example in the case of the A2-supertype, where most alleles are associated with the A2 serological antigen. In other cases the relationship is more complicated, such as the gene conversion relationship between the A2, A3 and A68 antigens. This latter example is somewhat of the exception that proves the rule. Supertype associations are based on shared binding specificity, which may result from both common ancestry and convergent evolution [110]. Thus, while alleles within a supertype may have a close evolutionary relationship, that is not a given. Also, alleles (supertypes) sharing specificity at one anchor position may be associated with very disparate specificities at the other.

Other groups have also utilized various methodologies to define supertypes. In general, our classification is in agreement with those derived by other approaches, as compiled by Hertz [32], Lund [23] and Tong [31]. This is not surprising given the good agreement observed between our initial dataset and other classifications, and that the methodology utilized here is not different from the one utilized to derive the original assignments. If there are variations, they usually represent the splitting of a supertype, or reassignment of individual alleles. As in any classification problem of this kind, there is no absolute truth in supertype assignments. The practical application of supertype classification schemes to identify degenerately binding peptides will ultimately show what classification scheme has the most practical value.

## Conclusion

The present study represents an update to the HLA class I supertype classification originally described almost a decade ago. Using MHC peptide binding motif data and MHC sequence information that has since become available, supertype associations have now been provided for over 750 HLA-A and -B alleles. In addition, the approach utilized has been made more transparent, allowing others to utilize the classification approach going forward.

## Methods

### MHC sequences

The sequences of HLA A and B class I alleles were obtained from the IMGT/HLA Database [91,113], release 2.9. Alleles with incomplete sequences were removed from further analysis. The residues forming the B and F peptide binding pockets were aligned as described in the Results section.

### Peptide binding motif reference panel

The main anchor peptide binding motifs recognized by HLA-A and -B molecules were compiled from our own data, or as reported at the SYFPEITHI database [87]. The HLA supertype associated with each motif was assigned as defined previously [11,12], and/or on the basis of the broad chemical specificity of each supertype indicated in Tables 1 and 2.

### Pocket analysis

The residues forming the B and F pockets of each allele in the motif reference panel were compiled as a lookup table in Microsoft Excel. Pockets were defined more stringently by considering all residues forming the pocket, or less stringently by considering only a subset of residues, denominated as key residues, hypothesized to be most directly involved in peptide binding. To assign a B and F pocket specificity for each of the remaining alleles, the reference panel was scanned to identify exact pocket sequence matches. If an exact match could be identified, the allele was assigned the corresponding B or F pocket specificity. Supertype assignments were then made by matching the B and F pocket specificity pattern with the supertype descriptions indicated in Table 2. If an exact match for either the full pocket sequence or key residue sequence could not be made at both the B and F pockets, the allele was considered unassigned.

## Abbreviations

EBV (Epstein-Barr virus); HBV (hepatitis B virus); HCV (hepatitis C virus); HIV (human immunodeficiency virus); HLA (human leukocyte antigen); HPV (human papilloma virus); IEDB (Immune Epitope Database); LCMV (lymphocytic choriomeningitis virus); MHC (major histocompatibility complex); SARS (severe acute respiratory syndrome).

## Authors' contributions

JS performed the sequence analyses, determined the supertype assignments and drafted the manuscript. BP participated in the conceptualization and design of the study, and assisted in the preparation of the manuscript. NF and CB assisted in the study design and data interpretation. AS participated in conceptualization of the study and its design, provided interpretation of the data, and helped to draft the manuscript. All authors read and approved the final manuscript.

## Supplementary Material

Additional file 1 HLA supertype classification of HLA A and B class I alleles. This table provides a complete listing of the HLA A and B alleles examined in the present study. The table is sorted in alphanumeric order to facilitate searching for a specific allele. For each allele, the supertype assignment is indicated, and it's corresponding B and F pocket sequences are shown. Click here for file
